# Determinants, Health Problems, and Food Insecurity in Urban Areas of the Largest City in Cape Verde

**DOI:** 10.3390/ijerph13111155

**Published:** 2016-11-22

**Authors:** Isabel Craveiro, Daniela Alves, Miguel Amado, Zélia Santos, Argentina Tomar Fortes, António Pedro Delgado, Artur Correia, Luzia Gonçalves

**Affiliations:** 1 Unidade de Saúde Pública e Bioestatística and Global Health and Tropical Medicine, GHTM, Instituto de Higiene e Medicina Tropical, IHMT, Universidade Nova de Lisboa, Lisboa 1349-008, Portugal; 2 Unidade de Saúde Pública e Bioestatística, Instituto de Higiene e Medicina Tropical, IHMT, Universidade Nova de Lisboa, Lisboa 1349-008, Portugal; daniela.alves@ihmt.unl.pt (D.A.); d2010058@ihmt.unl.pt (Z.S.); luziag@ihmt.unl.pt (L.G.); 3 Departamento de Engenharia Cívil, Arquitetura e Georecursos, Instituto Superior Técnico, Universidade de Lisboa, Lisboa 1049-001, Portugal; miguelpamado@tecnico.ulisboa.pt; 4 Instituto Nacional de Saúde Pública, Ministério da Saúde, Praia 719, Cabo Verde; argentina.fortes@insp.gov.cv; 5 Direção Nacional da Saúde, Ministério da Saúde, Praia 47, Cabo Verde; Antonio.Delgado@ms.gov.cv; 6 Comité de Coordenação Combate à Sida, CCS-SIDA, Ministério da Saúde, Praia 855, Cabo Verde; Artur.Correia@ccssida.gov.cv; 7 Centro de Estatística e Aplicações da Universidade de Lisboa, Lisboa 1749-016, Portugal

**Keywords:** food security, urban health, social determinants, Africa

## Abstract

Urbanization processes are intertwined with nutritional transition because there is easier access to food of low nutritional quality at reduced prices, changing dietary patterns and leading to an increase of non-communicable chronic diseases. This study aims to understand the perceptions for high blood pressure, obesity, and alcoholism, describing some interactions of these dimensions in the problem of food security in the city of Praia. A qualitative study was carried out under the framework of the research project “UPHI-STAT: Urban Planning and Health Inequalities—moving from macro to micro statistics”. Ten focus groups were conducted in three urban areas with distinct characteristics in the city of Praia, with a total of 48 participants. Participants reported frequent consumption of foods with poor nutritional quality, understanding the potential danger in terms of food security in the city of Praia. Easy access to and high levels of alcohol consumption, and poor quality of traditional drinks were mentioned by participants in the study areas. The impact of the economic situation on the possibility of access to safe and healthy options emerged as a differentiating factor.

## 1. Introduction

Food security implies promoting the right of all citizens to the regular and permanent access to food in sufficient quantity and quality [1].

In contrast, food insecurity is deeply linked to poverty, affecting the weakest social groups, with the right to food being more limited, both in terms of access to social and security networks, and productive capacity (capital, land, and agriculture products) [2]. There is evidence that families’ food insecurity, which relates to the financial ability in the household to have access to adequate food, is one of the markers of nutritional vulnerability [3,4].

Urban food insecurity is an emerging challenge, which is exacerbated by factors such as climate change; low income, which is the main cause of urban food insecurity; precarious living conditions; local environmental risks; and limited access to markets [5]. 

More than half of the world’s population lives in an urban context [6]. According to the latest official Census in Cape Verde, in 2010, 66.7% of the population lived in urban areas, mostly in the capital of the country (city of Praia) [7].

Problems of food security in Cape Verde are recurrent due to weak or irregular rainfall, resulting in water scarcity and drought cycles. In terms of food, the country is very dependent on the foreign markets, with low production (mainly family or subsistence type) [8]. In general, imported products are sold in shops and domestic production is sold in local markets and by street vendors. 

In developing countries, the processes of urbanization and nutritional transition can occur in parallel, namely due to easier access to food at low prices and low nutritional quality. In general, this type of food is rich in complex carbohydrates, fat, sugar, and cholesterol—and low in fiber and vitamins [9,10].

Dop et al. [11] evaluated the food consumption of families in the context of nutritional transition in Cape Verde, with the following results: (a) higher consumption of animal products in families with overweight and obese members, compared to families with members who had low birth weight; (b) a low consumption of fruits and vegetables in both types of families. According to these findings, in Cape Verde, energy availability is not a concern, however, nutrients seemed to be a problem in terms of food and nutrition security. 

According to data published by the World Health Organization (WHO) [12] on the profile of non-communicable diseases in Cape Verde, in 2013, 69% of deaths were due to non-communicable diseases, with a percentage of 35% for cardiovascular disease. It is estimated that 47.7% of men and 38.4% of women aged more than 25 years suffer from hypertension [12]. The prevalence of overweight and obesity in the country was estimated at 44% [12]. 

Studies indicate an association of food insecurity with noncommunicable chronic diseases, such as, hypertension and diabetes [13,14,15,16]. The relationship between food insecurity and obesity at the individual, family, or community level is not yet properly established [17]. Several studies suggested a positive relationship between food insecurity and obesity, despite some contradictory results [17,18,19,20]. This relationship appears to be more consistent in females [17,21,22,23,24].

For a long time, the relationship between high alcohol consumption and disease has been established. Rehm et al. [25] presented a systematic literature review in order to determine causality. Findings pointed out causality between alcohol and many chronic and acute diseases and injuries. Additionally, the authors mentioned the lack of studies on the effects of quality of alcohol. Other studies established the association between chronic ethanol consumption and an increased incidence of hypertension and an increased risk of cardiovascular diseases [26,27,28]. The burden of disease linked to alcohol may be exacerbated by informally and illegally produced beverages, as pointed out by WHO [28]. The objective of this paper was to analyze the perceptions of the participants about the health problems mentioned above (e.g., hypertension, obesity, and alcoholism), exploring potential links with food safety issues in the city of Praia.

## 2. Materials and Methods

### 2.1. Study Design Overview

We conducted a qualitative study using focus groups as a technique of data collection, carried out in three areas of the city of Praia, with different characteristics in terms of physical and social environments (formal, informal, and transition). The present study is included in the research project UPHI-STAT “Urban Planning and Health Inequality—moving to the macro for micro statistics” (PTDC/ATP-EUR/5074/2012), funded by the Foundation for Science and Technology (Portugal). The formal area (Plateau) presents an urban planning, with infrastructure and basic services provided. The informal area (part of the neighborhood of Vila Nova) developed without urban planning, having no infrastructure and adequate basic services created several problems, for example, in terms of sanitation and water supply. The transition area (part of the neighbourhood of Palmarejo) presents mixed features of formal and informal areas. The UPHI-STAT project started with a quantitative component [29], followed by a qualitative component which aims to complement and understand some aspects that emerged from the quantitative approach, seeking in depth comprehension of the results.

### 2.2. Sampling Procedures

In the first stage of the UPHI-STAT project, a probabilistic sampling strategy was used through a random selection of geographic coordinates as described by Gonçalves et al. [29]. A questionnaire was applied (n = 1912) by local interviewers and participants were asked to: (1) provide a contact for future participation in the qualitative study; and (2) to carry out an assessment of nutritional status by local nutritionists (second stage, n = 599). From the initial set of participants who gave their telephone contacts (n = 413), 144 fulfilled the first and second stages of UPHI-STAT (Table 1). The opportunity to participate in the qualitative study was given to these 144 participants, with three contact attempts by telephone. At the end, 48 individuals participated in this qualitative study. The remaining 96 participants did not respond, despite the initial willingness to participate, they did not attend or they said they were not available (Table 1).

From the findings of both stages—questionnaire and assessment of nutritional status—we created the focus groups according to the following characteristics: (1) belong to one of the studied urban areas; (2) the sex of the participant; (3) age: 18–40 years and ≥41 years; (4) body fat (%): —for women underfat or healthy (<24%), overfat or obese; (≥24%)—and for man underfat or healthy (<16%), overfat or obese (≥16%), according to previous studies [30].

### 2.3. Data Collection and Analysis

The qualitative study was carried out between October and November 2014. All participants provided written informed consent before study participation. Moderation of the focus groups was under the charge of a Cape Verdean sociologist resident in Praia and fluent in Creole (native language). Co-moderation was ensured by two researchers of UPHI-STAT project. The moderator was previously trained by the lead author. 

Focus groups were undertaken in convenient quiet locations. Focus groups, consisting of 3–8 participants, were led by a topic guide based on the objectives of the study and existing literature. It focused on questions that explored perceptions about health topics—hypertension, overweight, and obesity—and the links with food, dietary habits, and alcohol consumption (Table 2).

Focus groups lasted between 30–50 min. All focus groups were audio recorded and transcribed verbatim by a Cape Verdean fluent in both Creole and Portuguese. The transcripts were reviewed and verified by members of the research team who speak Creole. 

Transcripts were analyzed manually using thematic content analysis, with both inductive and deductive coding, to identify emerging themes guided by a template approach [28]. A provisional template was created based on deductive themes which were broad and relevant to the study questions and current literature [28]. Two researchers (IC and DA) independently read the transcripts, applied this template to a subset of the data, and discussed the coding scheme and emerging themes. A revised template was then applied to all transcripts. As coding proceeded, additional themes emerged. 

### 2.4. Ethical Considerations

The UPHI-STAT project was approved by National Committee for Ethics in Research for Health (Doc. N.52/2013), Cape Verde, and Ethics Council of IHMT (Doc n°. 24-2013-PI), Portugal. Written informed consent was obtained from all participants. Confidentiality and anonymity were ensured. 

### 2.5. Characteristics of Participants 

From 10 focus groups, a total of 48 residents of the three urban areas (16 men and 32 women) participated in the study. Mean age of participants was 39.75 (15.90) years. 41.7% of participants reported having secondary education. The percentage of participants with no education and preschool education (16.7%) is equal to the percentage of participants with high school education. Only 25.5% had a partner and 77.1% reported having children (Table 3).

## 3. Results

### 3.1. Health Problems: Hypertension and Obesity—Perceptions about the Main Causes

While exploring the causes of hypertension, eating habits (considered not always balanced) and characterized by an extreme consumption of unhealthy foods—salt, fat, fried foods, sugar, and the stress of daily life, emerged as important issues in the discourses of men and women of the focus groups in the three study areas, as exemplified in the following citations.

*“The causes* [of hypertension] *are exaggerated consumption of salt and fatty foods (such as pork and beef)”.*

(FG-F1-women)

“Unemployment causes stress and ... people who are studying, when you finish college and cannot find work. This causes a lot of stress”.

(FG-T7-men)

*“Stress, excessive intake of salt, alcohol* [are all causes of hypertension]*”*.

(FG-I2-men)

“As my colleagues just talk, is not only the food, also the concerns ... with regard to food, we know we have to avoid many things that are bad like salt. I just eat something with lots of salt, then I feel the difference. It’s salt and fat”.

(FG-I6-women)

The stress that affects city residents was also pointed out.

Women in the informal area and men in the transition area also highlighted the stress caused by the unsafe environment in the neighborhood, alongside unemployment and single parenthood as main factors of hypertension.

“*There are many people here in Cape Verde with this problem* [of hypertension] *because of stress, we are living in an insecure environment”.*

(FG-I5-women)

“Insecurity also causes a lot of stress at home, when we turn on the TV. (...) Cape Verde has many single parents and it also hurts a lot because only one person is working to support the house, and gets a lot of problems in the head, I think it also influences.”

(FG-T7-men)

But only women in the informal area mentioned the stress caused by difficult economic situations and obesity as causes of hypertension.

“(…) There is a lot of unemployment, crime, too much tension, the economic situation is bad and there is also very excessive consumption of salt, obesity, all this causes hypertension”.

(FG-I5-women)

Women in the transition area reported the family history and the coexistence of other chronic diseases, for example diabetes, as specific causes of high blood pressure, as illustrated in the following sentence.

“*Well, first I think it’s the food ... Another is the family history, for example, in my case, my mother is hypertensive, my grandmother, my aunt ... and I also have a tendency, whenever I am anxious my tension starts to rise .... eating habits because of the rush, lifestyle, and family history”.*

(FG-T9-women)

And only men in the informal area talked about alcohol, drugs, depression, and general concerns of daily life, referring them as reasons for blood pressure and hypertension.

“There are many young people now with high blood pressure because of drug use, alcohol, stress”.

(FG-I2-men)

When participants talked about the factors of overweight and obesity in the three areas, both women and men mentioned the changes in dietary patterns, due to imported habits (e.g., introduction of fast food), losing habits of consuming “local products”, particularly due to the scarcity of such products. Lack of physical activity was also unanimously discussed.

“But from the moment we begin to eat imported foods.—Yes, precisely. When we ate natural foods, ‘product of our land,’ no one had this problem. Look at the example of my father who is 96 years old

*(…)—I think they are* [changing us all]*".*

(FG-I4-men)

*“I think what leads to overweight is poor diet, especially when they do not include salad, fish, fruit and also the lack of physical exercise, drink*[ing] *less water (...)”.*

(FG-I5-women)

Men in the transition area reinforced the idea of a gradual trend among the residents of the city of Praia to replace local products with imported products, and that constitutes one of the reasons why people are unhealthy.

*“Because the food we now consume is not from our land as two years ago. Two years ago we were eating only products of agriculture* [and] *fishing and today we have almost no fish because the boats are coming out to fish the best fish and take it. We have few fish and it is expensive. And we do not have access, then the only solution we have is ... and the food as we know has many chemicals that bring diseases such as cancer and some more (...) "*.

(FG-T7-men)

Family and/or cultural habits were also mentioned in the three areas, for both women and men, as a dominant factor to unhealthy options. According to participants, there is a greater concern to eat in quantity and less about the quality, as illustrated by the following citations.

“[Traditionally] *We seek more quantity than quality”*.

(FG-T7-men)

*“The problem is that the Cape Verdean*[s] *like food that fills the stomach, the salad does not satisfy enough. —Salad is a light food, does not satisfy, you can eat now and get it over with hunger. In Cape Verdean tradition, it is not so. —Yes, exactly, the Cape Verdean prefers cachupa* [a traditional dish] *with chicken, fish, chorizo with everything and anything”.*

(FG-I2-men)

“We Cape Verdeans always prefer a plate of "cachupa” (…)—“Beans with fried fish (preferably). Because the food is greasy, has too much salt and almost always includes fried foods (...)—It’s a bomb to the stomach”.

(FG-I5-women)

The availability of street food because of the high levels of salt, fat, and sugar, was considered unhealthy, by men and women in the informal area.

*“You can eat well in kiosks, for example, I have eaten there, and they usually have salads with carrots, beets, lettuce, and cucumber. Where I do not advise, it is “Sucupira”* [local market] *... Because the food they make there is harmful to health (...) too fat”.*

(FG-I5-women)

*“Cachupa, bean stew with meat, fish, all mixed up, I do not think that is healthy ..."* (street food).

(FG-I2-men)

Only women in the three areas spoke about the problem of spending too many hours without eating due to lifestyle (lack of rest and sleeping hours) and they reported difficulties with respect to meal times. The number of meals varied between three and four by day, not corresponding to the dietary recommendations adopted in Cape Verde [31].

*“We* [take our] *morning coffee (...) our routine does not allow us to eat on time. (...)—I agree ... our routine does not have a power on time. (…)—In addition to not eat healthy foods Cape Verdeans have a bad habit of not eating on time”.*

(FG-F1-women)

*“In* [the] *poor area is breakfast, lunch and dinner in the VIP area they have lunch in the middle and we do not snack in between. That’s why we, the poor, we care to take a good coffee, sometimes with good bread with butter and a glass of milk. At lunch increases our concern to eat much for the belly is full. We* [eat] *rice, beans, or cachupa and that’s it. (…)—It’s the same thing. Once we lunched* [on] *rice or xerém and dinner was cachupa. Now things have changed, if you eat rice or cachupa at lunch, at dinner you can make a soup. And not all people do it because it depends on the economic possibilities of each one”*.

(FG-T10-women)

In the informal area, women pointed out factors affecting obesity, such as lack of time to cook, the preference for fried foods because they are tastier and the consequences of contraceptive methods.

“There are people who do not have time to cook because of the work routine, but some eat because they like, you know everything that is fried tastes different (…)—It is not only the food which increases weight, contraceptive method also contributes to it ... This is my case”.

(FG-I5-women)

Men in the informal area talked about the impossibility of eating healthy foods due to monetary conditions, e.g., raising the issue of forced options on the intake of less healthy food because it is the cheapest on the market and the most accessible, independent of the knowledge about healthy food options.

*“Yes ... (I think people do not eat well). (…)—I do not say good or bad, depends on the conditions of each one, sometimes for lack of economic conditions you cannot make a healthy diet. (…) —If you can only afford to eat in these places (restaurants/kiosks) you cannot do anything, so I think* [having] *a healthy diet depends on the economic conditions of each one ... For example, if I have $70* (Cape Verde escudos ($); $100 = 0.90€ (euros)—currency in October 2016) *and find a meal in these places (cachupa, beans ...), even aware that it is not appropriated, and it is not good for our health, I will eat”*.

(FG-I3-men)

Low levels of physical exercise and progressively sedentary lifestyle were mentioned as causes of hypertension and obesity, by all participants in focus group discussions. The following citation is one example.

*“I also think that the lack of physical exercise can lead to those problems too* [obesity and hypertension]*...* ”.

(FG-I3-men)

When asked about the reasons why residents in their neighborhoods register low levels of physical activity, the participants said that, on the one hand young people due to unemployment and insecurity of the city, and on the other single parents with family responsibilities have an excessive burden which hampers the physical activity.

Study participants were asked to talk about the people they considered as eating worse and the reasons why, based on their experiences. In the three areas, both men and women considered street and market vendors, people working in offices, and taxi and bus drivers as the most vulnerable groups for obesity. The following reasons were cited: 

(1) They have more sedentary lives;

*“People who work sitting (...).—Vendors* [Rabidantes]* (...).—The people working in the market”.*

(FG-I2-men)

*“People who work* sitting [are] *more likely to get fat ... For example, people working in offices”.*

(FG-I3-men)

“Students, workers, taxi drivers, those working in the bus sector (…)—They eat a lot of food “on the street" (…).—Because they always work in the same place and do not have time to prepare the food”.

(FG-T7-men)

(2) They have more limited food options, mostly unhealthy. Essentially, because these professional groups are exposed to ‘street food’ availability (considered mainly unhealthy), and their work environment was described as a ‘factor for obesity’.

(Market vendors)*—Yes, they also tend to put on weight, they are still and always eating fatty foods”.*

(FG-I3-men)

(Market vendors)*—They have poor eating habits, such as fried food, street food”.*

(FG-T8-women)

*“There are people who work on the street and do not have time to go home.—They have to eat at the kiosks.—Certainly* [it’s] *not the cheapest food, but it’s a matter of time”.*

(FG-I6-women)

### 3.2. Health Issues: Alcohol, Quality, and Quantity—A Double Problem?

When participants were asked to express their perceptions about alcohol consumption in the city of Praia, the quality of alcoholic beverages, the quantity of alcohol consumption, and the availability of alcoholic drinks, they considered all dimensions as serious problems.

#### 3.2.1. Quality of Alcoholic Beverages 

In Cape Verde, there is a recognized tradition of production and consumption of alcohol derived from sugar cane (brandy, commonly known as “grog” and “punch”). The issues of quality of alcoholic drinks emerged during the focus group discussions, in the three areas, and both men and women pointed out that grog and other drinks (e.g., beer) were ingested in large quantities in the city of Praia.

According to participants, men and women in the three areas, there are situations where grog is handled without quality control, which is a situation of food insecurity, and participants could realize the potential negative impact on health outcomes.

“—There is no control of production quality”.

(FG-F1-women)

“(...) the quality of ‘grog’ is very bad and damages health”.

(FG-I5-women)

“It’s easy to produce the grog”.

(FG-I6-women)

As illustrated in the next citation, when men in the transition area talked about the quality of grog they pointed out the *“domestic and uncontrolled way of* [producing] *the beverage”.*

“—Also the quality of alcohol (…)—People put water and make “grog” in the frying pan…”

(FG-T7-men)

#### 3.2.2. Quantity of Alcohol Consumption 

According to the generalized perception of the study participants, grog and other alcoholic drinks are ingested in great quantity. All participants in the three areas (both men and women) agreed on the extent of the problem of alcohol consumption, considered “too much” by the women during the focus group discussion in the formal area.

“[collective answer] *A lot of* [alcohol] *consumption*!”

(FG-T7-men)

“Question**—**What do you think about the consumption of alcohol in the city? —*It´s serious!* [all the others agreed]”

(FG-I6-women)

The alcohol intake is very common: “*there is no difference between weekday and weekend*”, as stated from both men and women in the focus groups discussions in the informal area.

“It’s more or less the same (…) is equal consumption on weekdays and at the weekend...”

(FG-I2-men)

*“But now for young people* [it] *is not like that ... they drink every day”.*

(FG-I4-men)

*“In my area is every day (...). Serious!* [Alcohol intake]”

(FG-I6-women)

Drinking is generalized to all parts of the city and social groups and events (e.g., dancing clubs, festivals, street and family parties). It was reported by all participants, regardless of area of residence, outlining the problem as “widespread” throughout the city.

“Both ... Family parties and street events”.

(FG-I5-women)

“Drink in all areas”.

(FG-T7-men)

Young people were considered as the group that presents higher intakes, which, according to the general perception of the participants in the three areas start in a very early age, as exemplified in the following citations reporting the dialogues in some focus group discussions.

“Most young people now;—Young people drink more;—More the younger than the older ones.

*Even young people, even children;—10 to 12 years (...)”*.

(FG-I2-men)

“In my neighborhood are mostly young people who consume alcohol (...)”.

(FG-I3-men)

*“Usually start from 11 to 16 years (…)—When I was in high school we took juice for lunch, but now is the ’bottle’ (grog) $100* ($100 = €0.90—currency in October 2016)*”.*

(FG-T8-women)

#### 3.2.3. The Availability of Alcoholic Beverages

When discussing the type of consumption and which groups they consider having higher alcohol intakes in the city, most participants (both men and women in the different groups) mentioned problems related to the access to alcohol by children, and described the purchase of alcohol by minors. Despite the existing Law n° 27/V/97 *“prohibiting the sale of alcoholic beverages to minors and advertising of alcoholic beverages in some situations”*, revised in 2016 (Law n° 51/2016), as illustrated in the following citations:

“There are also those kiosks selling drinks to anyone, even to minors because there is no supervision. (…) There are many people who sell alcohol and disguise that as they are selling candy”.

(FG-F1-women)

“It is normal to see a kid with a bottle of alcohol, sometimes even out of curiosity (...).—It’s not limited (access of alcohol to children), although there is a law that prohibits the selling of alcoholic beverages, there are places that sell to children simply because they say were sent by the parents (...)”.

(FG-I2-men)

During the focus group discussions, participants in the three areas, both men and women, mentioned two interrelated topics that constitutes a potential problem: 

(1) The negative impact of low prices of alcoholic drinks.

“I think the liquor is cheaper than food”.

(FG-I2-men)

*“Of course (the drink is cheaper than food). 1 L of spirits costs 200 escudos* ($200 = €1.80—currency in October 2016). *(…) It’s not cheap. It’s almost free!”*

(FG-I4-men)

*“Now you can buy a bottle of brandy for $100* ($100 = €0.90—currency in October 2016). [Another woman agreed]—*$90*

(FG-F1-women)

(2) Easy access to alcoholic beverages, which includes the possibility of young children to buy alcoholic drinks, as stated in the citations below.

“Let me just say one thing, it’s easier to raise a seller from bed in the middle of the night to sell a bottle of alcoholic beverage than 1 kg of rice”.

(FG-I2-men)

“Now I can send my son to buy beer and he can buy”.

(FG-I6-women)

One feature highlighted by participants in the three areas, was the influence of the "example" of adults, in terms of exposure of children at very early ages to alcohol consumption.

“This is because children tend to follow the examples of those closest and nowadays all kids from 10 to 20 years consume alcohol, I know cases of children from 11 to 15 years…who consume alcoholic beverages, smoke and there’s nobody to guide them (...)”.

(FG-I3-men)

## 4. Discussion

This qualitative component of a mixed method study offers critical insight into some of the contextual factors affecting hypertension, obesity and overweight, and alcohol consumption in Praia, based on the participant’s perceptions.

The findings are aligned with existing research on the influences on hypertension, which are similar to the causes of health problems reported by participants in the focus group discussions (e.g., the type of eating habits, changes in dietary patterns, low level of physical activity) [9,10,13,14,15,29].

When we explored our data according to the area of residence and gender, we concluded that there are similarities between the study areas and gender in terms of the main causes of hypertension: unhealthy food consumption (e.g., salt, sugar, fat) and the stress of daily lives. Women and men in the informal area brought to discussion the influences of the stress caused by unsafe environment, unemployment, single parenthood. Women in the informal area specified the stress triggered by the economic difficulties.

Women in the transition area are aware of the importance of family history and chronic diseases as diabetes and hypertension.

Men in the informal area mentioned alcohol and drug intake and depression as specific causes of hypertension.

There is evidence that sodium intake above the recommended (eating habits), body weight, and possibly stress may be important predictors of development of hypertension [32,33,34,35]. The association between food insecurity and hypertension, even after adjustment with sociodemographic characteristics, was described by several authors (e.g., [14,15]). Through analysis of a 24-h dietary recall applied to the quantitative study of the project UPHI-STAT it was concluded that participants presented fiber and micronutrient deficiencies and lipid and sodium excess (data under publication).

As mentioned earlier, according to Ng et al. [36], about 44% of Cape Verdean presented overweight and obesity, based on self-reported weight and height. The UPHI-STAT project found percentages of 35.6% for men and 42.0% for women in Praia city [29]. For women, the percentages of overweight and obesity varied among urban areas, with 43.5% in the formal area, 36.3% in the transition area, and 55.6% in the informal area [29].

When we explored the perceptions of the participants of focus group discussions, regarding the factors of overweight and obesity, the importance of changing dietary patterns was consensual (e.g., inclusion of fast food). Cape Verde is in a nutritional transition period that is characterized by the consumption of high fat, refined carbohydrates, cholesterol, and sugar [10] and low consumption of fruits and vegetables [11]. According to Food Agriculture Organization (FAO), among women, nutritional transition is causing a rapid and significant increase in the prevalence of overweight and obesity [37].

In all focus group discussions, the lack of physical activity, and family and cultural habits were pointed out as causes of overweight and obesity. Men in the informal area detailed the impossibility of eating healthy food because of the economic situation, even when people are informed. Thus, our results highlight the economic condition as a differentiating factor to buy healthier food in the informal residential area, even when participants are aware of the “correct” nutritional choices. Which is in line with other studies that demonstrate the families’ nutritional vulnerability due to financial inability to purchase adequate food [2,38].

Another issue raised by women in the informal area was the lack of time to cook and subsequent intake of unsafe street food. These barriers were often described in other type of studies and in other contexts [39,40].

Food insecurity and obesity can be caused by many factors, including low level of income and the consequent restriction on the access to food with higher nutritional quality, as well as the adoption of more sedentary lifestyles, with consequent imbalance between consumption and energy expenditure [41]. Taxi and bus drivers were described as eating worse because of sedentary lifestyles and higher ingestion of caloric street food by the participants in our study, which is in line with other studies that included this professional group in higher risk of obesity, although in a different context [42].

The production of grog is overseen with special attention by government. In Cape Verde there is legislation (Decree-Law No. 89/92), establishing the general rules for the control of quality of food which is produced in the country or imported. There are several entities to carry out this control with overlapping competencies and lack of coordination between them [43].

Vieira et al. [8] studied the quality and safety of traditional foods in Cape Verde including grog and they found nonconformities in the manufacturing process and they acknowledge the need for corrective measures in order to improve the quality and safety of products. Our findings are in alignment with this previous research, suggesting that grog production is not sufficiently controlled.

Young people were considered the group that presents larger intakes, which—according to the general perception of the participants—began at a very early age. This view coincides with the results of the WHO report on alcohol consumption [28]. In Cape Verde, there is a cross-practice of different social classes and age groups, including children and young people (13–16 years) [44].

Our findings suggest the purchase of alcohol beverages by children, despite a specific regulation (law n. 271/V/97) prohibiting the sale and advertising of alcohol among minors [40]. Additionally, participants reported that since a very early age, children are exposed to adults’ alcohol consumption and there is evidence that parents with alcohol-related problems increase the risk of alcohol-related problems in their children [44,45]. Moreover, in a study in Africa, the authors pointed out that children living with parents who drink alcoholic beverages is one of the factors associated with the consumption of alcohol by students [46].

In 2010, a WHO report [38] on the alcohol consumption states that Cape Verde is the third largest consumer of the Community of Portuguese Speaking Countries (CPLP), with an average of 6.9 L of pure alcohol, being exceeded only by Angola, with an average consumption of 7.5 L of pure alcohol. The average consumption in the African region was 6 L of pure alcohol. These data confirm the perceptions of participants about alcohol consumption.

Strengths and limitations: the focus groups allowed us to gain a deeper understanding of Praia’s residents’ perceptions of the causes of hypertension, obesity, and alcohol consumption, due to the rich discussions. The separate focus groups for the three areas under study can represent an extensive range of opinions.

One of the major limitations of the study is that we could not assure a similar number of participants in the focus groups in the three areas, resulting in an absence of men and an under representation of women in the formal area, compared with other study areas. Additionally, the participants in the focus group were eventually the most interested, because they fulfilled all stages of the project and we cannot control the potential bias.

## 5. Conclusions 

The perceptions of the participants in the qualitative study point towards the existence of a link between the eating habits and sociocultural conditions. The difference is related to the impact of the economic conditions in the possibilities of healthier food choices, even when the participants have knowledge about appropriate food practices, especially in informal area that seems to be at disadvantage. This is a recognized marker of nutritional vulnerability.

In focus groups discussions, some problems associated with the type and feeding practices that have implications in terms of food were mentioned. The availability and consumption of the street food, which is easier to achieve, but presents quality problems—particularly those related to fat, salt, fried foods, and even lack of hygiene—which constitute potential dangers in the context of food security.

It is important to highlight the issues associated with alcoholic beverages, accumulating problems of quantities ingested (high consumption) and quality of the products consumed, according to participants. In particular, the products available on the market at very low prices, and ‘modified’ in terms of its production (becoming hazardous to health), were mentioned by study participants.

The problems identified in the qualitative study are related to certain professional groups, in particular those which have the greatest vulnerabilities in terms of food security due to the type of food they eat, representing a triple negative burden—less healthy, lower quality, and less safe.

This situation enhances the importance of acting on the social determinants in an urban space in order to ensure better conditions with potential socio environmental positive impact on health outcomes of the residents of the city of Praia.

## Figures and Tables

**Table 1:** UPHI-STAT participants.

**Stage 1 (Questionnaire UPHI-STAT)**				
**Participants and Focus Groups by Area**	**Formal Area (n)**	**Transition Area (n)**	**Informal Area (n)**	**Total (n)**
Participants (total)	145	1444	623	1912
Participants who gave the contact for qualitative study	31	253	129	413
**Stage 2 (Nutritional Status Assessment)**				
Participants (total)	22	283	294	599
Participants contacted	6	78	60	144
**Qualitative Study**				
Number of focus groups	1	4	5	10
Focus group identification	FG-F1-women	FG_T7-men FG_T8-women FG_T9-women FG_T10-women	FG_I2-men FG_I3-men FG_I4-men FG_I5-women FG_I6-women	
Participants	3	24	21	48

**Table 2:** Questions included in the topic guide.

Subjects	Question
Health issues	Hypertension: What are the causes of hypertension?
	Overweight and obesity: What are the factors that lead people to be overweight or obese? When referring eating habits and physical activity: According to your experience, why some people do not eat healthy foods or do exercise as often as they should?
	Alcohol: What is your perception about alcohol consumption in Praia?

**Table 3:** Demographic characteristics of focus group participants.

Participants	Statistics
Gender, n (%)	
Men	16 (33.3)
Women	32 (66.7)
Age (years), mean (SD *)	39.75 (15.90)
Academic qualifications, n (%)	
None and preschool	8 (16.7)
Primary	12 (25.0)
Secondary	20 (41.7)
High school	8 (16.7)
Marital Status, n (%)	
Without partner	35 (74.5)
With partner	13 (25.5)
Children, n (%)	37 (77.1)
Body fat classification, n (%)	
Underfat or healthy	14 (29.2)
Overfat or obese	34 (70.8)

* SD—standard deviation.
